# General practitioners’ views on cardiovascular prevention for ethnic minorities—a qualitative study in the Netherlands

**DOI:** 10.1093/fampra/cmad030

**Published:** 2023-03-30

**Authors:** Joshua A N van Apeldoorn, Alet K Roozekrans, Ralf E Harskamp, Edo Richard, Charles Agyemang, Eric P Moll van Charante

**Affiliations:** Department of Public and Occupational Health, Amsterdam UMC, University of Amsterdam, Amsterdam Public Health Research Institute, Meibergdreef 9, Amsterdam, The Netherlands; Department of General Practice, Amsterdam Public Health Research Institute and Amsterdam Cardiovascular Sciences, Amsterdam UMC, University of Amsterdam, Amsterdam, The Netherlands; Department of General Practice, Amsterdam Public Health Research Institute and Amsterdam Cardiovascular Sciences, Amsterdam UMC, University of Amsterdam, Amsterdam, The Netherlands; Department of General Practice, Amsterdam Public Health Research Institute and Amsterdam Cardiovascular Sciences, Amsterdam UMC, University of Amsterdam, Amsterdam, The Netherlands; Department of Public and Occupational Health, Amsterdam UMC, University of Amsterdam, Amsterdam Public Health Research Institute, Meibergdreef 9, Amsterdam, The Netherlands; Department of Neurology, Donders Institute for Brain, Cognition and Behaviour, Radboud University Medical Centre, Nijmegen, The Netherlands; Department of Public and Occupational Health, Amsterdam UMC, University of Amsterdam, Amsterdam Public Health Research Institute, Meibergdreef 9, Amsterdam, The Netherlands; Department of Medicine, Division of Endocrinology, Diabetes and Metabolism, The Johns Hopkins University School of Medicine, Baltimore, MD, United States; Department of Public and Occupational Health, Amsterdam UMC, University of Amsterdam, Amsterdam Public Health Research Institute, Meibergdreef 9, Amsterdam, The Netherlands; Department of General Practice, Amsterdam Public Health Research Institute and Amsterdam Cardiovascular Sciences, Amsterdam UMC, University of Amsterdam, Amsterdam, The Netherlands

**Keywords:** cardiovascular disease, cardiovascular risk factors, culturally sensitive care, ethnic minorities, general practice, prevention

## Abstract

**Objectives:**

While ethnic minorities in Europe are disproportionally affected by cardiovascular disease (CVD), little is known about how general practitioners (GPs) perceive differences in risk or care needs across ethnic minority groups. Therefore, we explored GPs’ views on whether ethnicity influences cardiovascular risk, whether a culturally sensitive approach is warranted, on potential barriers in the provision of such care, and to find potential opportunities to improve cardiovascular prevention for these groups.

**Methods:**

We conducted a qualitative study by interviewing GPs practising in The Netherlands. The interviews were semistructured, audio-recorded, and analysed by 2 researchers using thematic analysis.

**Results:**

We interviewed 24 Dutch GPs (50% male). GPs’ views on the impact of ethnicity on CVD risk varied widely, yet it was generally recognized as a relevant factor in cardiovascular prevention for most minority groups, prompting earlier case-finding of high-risk patients. While GPs were aware of sociocultural differences, they emphasized an individualized approach. Perceived limitations were language barriers and unfamiliarity with sociocultural customs, leading to a need for continuing medical education on culturally sensitive care and reimbursement of telephone interpreting services.

**Conclusion:**

Dutch GPs have differing views on the role of ethnicity in evaluating and treating cardiovascular risk. Despite these differences, they emphasized the importance of a personalized and culturally sensitive approach during patient consultations and expressed a need for continuing medical education. Additional research on how ethnicity influences CVD risk may strengthen cardiovascular prevention in increasingly diverse primary care populations.

**Key messages**

- GPs’ views on the impact of ethnicity on CVD risk and treatment varied substantially.
- GPs emphasized an individualized approach within patient’s sociocultural context.
- GPs expressed a need for continuing medical education on culturally sensitive care.

## Introduction

Cardiovascular disease (CVD) is the leading cause of death worldwide.^1,2^ In Europe and North America, ethnic minority groups are disproportionally faced with CVD and its risk factors, often starting at a younger age than the host populations.^3–9^ For instance, in the Netherlands, the prevalence of hypertension among groups of African descent is 1.5–2 times higher, and the risk of diabetes is 3–4 times higher than the host population.^9,10^ Explanations for these differences include biological/genetic factors and differences in lifestyle, access to health care, socioeconomic status (SES), cultural and health beliefs and preferences, and ethnic discrimination.^11–13^ As most CVD risk factors are amenable to change; preventive care is important to address this health disparity.^14,15^ General practitioners (GPs) play a key role in identifying at-risk patients who could benefit from preventive measures,^16^ as they have a longstanding relationship with their patients and, often, their family members, facilitating the long-term provision of health behaviour advice and counselling.

Despite substantial evidence of ethnic health disparities, most CVD guidelines refrain from providing ethnic-specific directives. Previous research among Dutch GPs indicated that providing cardiovascular prevention for ethnic minority groups might be challenging due to cultural differences affecting lifestyle and medication compliance.^17^ Likewise, research among African migrants residing in Amsterdam indicated that their perceptions and preferences on health and treatment might differ from the biomedical perspective.^18,19^ The combination of increased CVD risk burden and communication difficulties challenges optimal preventive care for ethnic minorities, but little is known about the perspective of GPs. Therefore, we aimed to explore GPs’ views on whether barriers exist in providing CVD prevention for these groups and whether they feel a culturally sensitive approach for ethnic minorities is warranted, and to find potential solutions to improve the quality of preventive care for ethnic minority populations.

## Methods

### Participants

We conducted interviews with 24 GPs in the Netherlands. GPs were initially contacted through our network and social media and subsequently through snowballing, in which we asked participating GPs to suggest colleagues who might view or approach this subject differently from theirs. Apart from the first 5 GPs, the participants had no prior connection with the interviewers, and none had a professional relationship with the interviewers. We used purposive sampling on years of experience, sex, migration background, practice location (urban versus rural areas with relevant numbers of ethnic minority inhabitants), and type of practice (solo/duo practice versus group practice. Over time, we approached GPs by email (*n* = 62) and telephone (*n* = 24) to reach a diverse sample. The GPs we approached per email were sent 1 reminder email if they did not respond to the initial correspondence within 2 weeks. Forty-seven (55%) did not respond to our invitation, and 15 (17%) declined participation. Reasons to decline were lack of interest to partake in an interview and lack of time. We based our sample size on whether we reached data saturation, defined as the point when we gathered enough data to understand the experiences and perspectives of the participants and no new themes were identified.^20^ Therefore, not all GPs willing to participate needed to be interviewed.

### Data collection

Between May 2021 and November 2021, 2 junior physician-researchers (JANvA, male/AKR, female) held semistructured interviews following an interview guide (Supplementary File 1). The interviewers and interviewees were aware of each other’s profession and level of experience. During the interview, we focussed on the potential influence of patients’ ethnic background on overall CVD risk, providing lifestyle guidance and treatment, the challenges in providing cardiovascular prevention, and the possible solutions to these challenges. This study focussed mainly on experiences with patients of Turkish, Moroccan, and Surinamese descent, as these are the largest migrant population groups in the Netherlands.^21^ We conducted the first 2 interviews together to establish concordance in approach. We iteratively adapted the interview guide during the data collection period. The duration of the interviews ranged between 30 and 70 min. No repeat interviews were deemed necessary. Given the restrictions imposed due to the COVID-19 pandemic, interviews were conducted through video conferencing (*n* = 21) and at GPs’ homes (*n* = 3). All interviews were audio-recorded with the interviewees’ permission and transcribed verbatim. Field notes were taken during all interviews. Following every interview, the GPs were asked for feedback about the research questions’ relevance and suggestions for additional questions to increase content validity. After the 3rd, 8th, and 20th interviews, the authors (JANvA, AKR, and EPMvC) extensively discussed the findings and potential key themes. Afterwards, the findings were discussed with the full research group.

### Coding and analysis

The authors performed thematic analysis following the phases set out by Braun and Clarke.^22^ Firstly, the 2 interviewers (JANvA and AKR) listened to the audio recordings and read the transcripts to become familiar with the data. Secondly, we independently derived initial coding trees from the interviews using the qualitative data software MaxQDA.^23^ Thirdly, we developed a coding system to generate a thematic map of main themes and subthemes. Fourthly, both interviewers reviewed the codes and refined the themes to reach consensus on potential themes. Fifthly, the core research group (JANvA, AKR, and EPMvC) repeatedly discussed this coding system until consensus about themes was reached. The themes and thematic maps were reviewed and refined. The collected data were re-read to ensure the thematic maps represented the data set. The resulting themes were refined, and the full research team considered and discussed a narrative of the main findings. Sixthly, using these fully worked-out themes, we produced our report. We selected illustrative extracts and translated the quotations from Dutch to English. We reported our study following the standards for reporting qualitative research: a synthesis of recommendations^24^ and the consolidated criteria for reporting qualitative research (COREQ) (Supplementary File 2).^25^

## Results

**Fig. 1.**
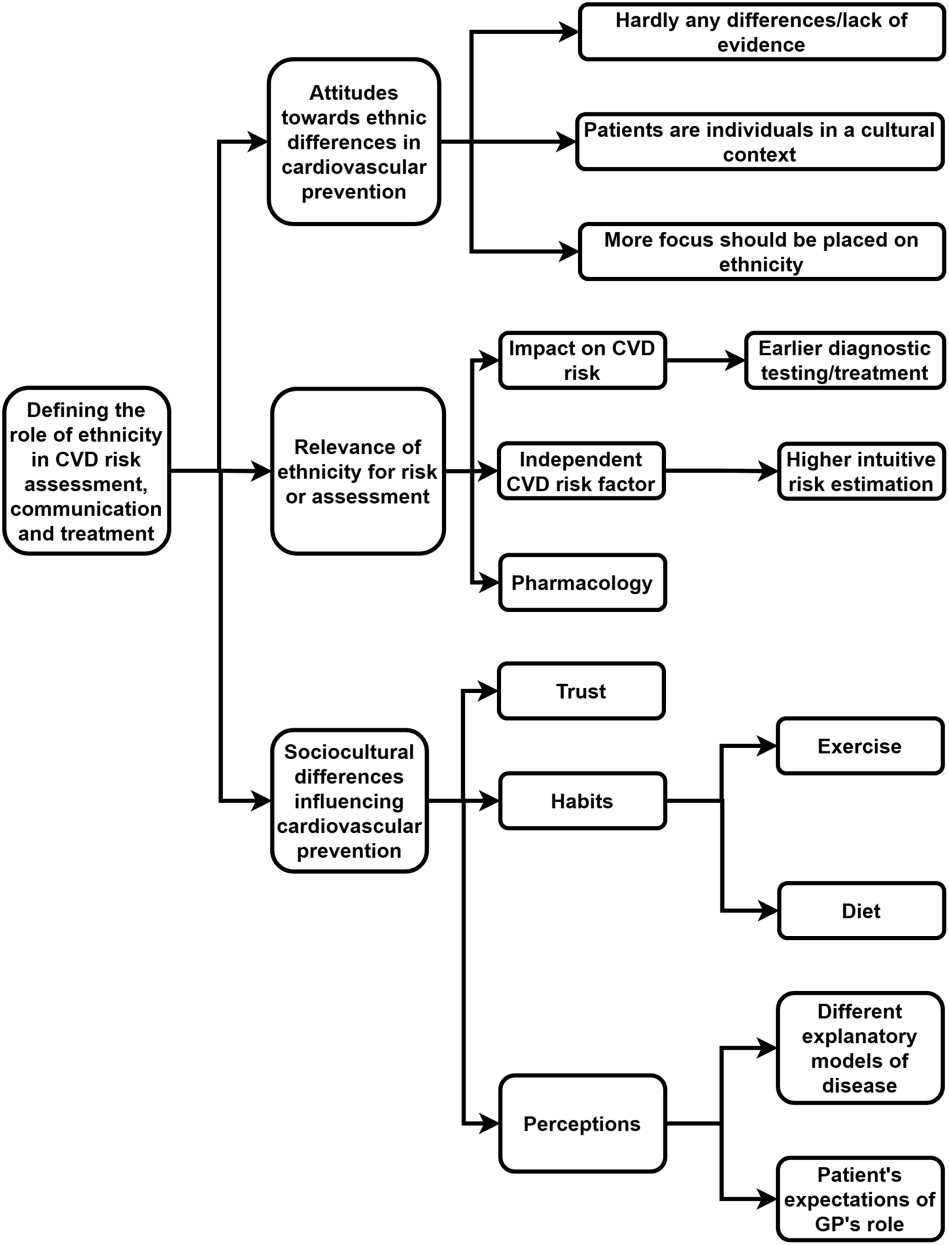
Thematic analyses key theme 1.

**Fig. 2.**
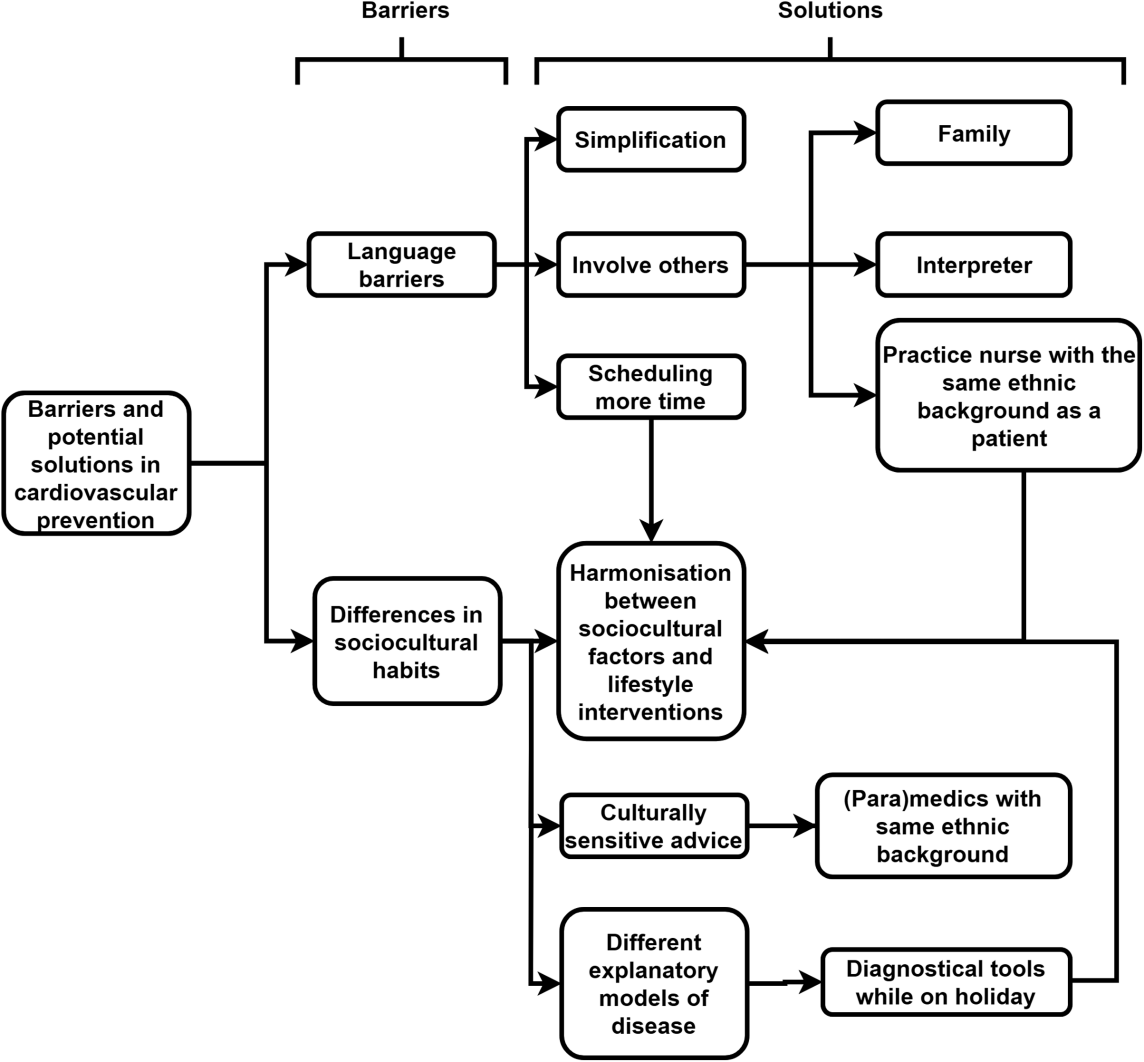
Thematic analyses key theme 2.

**Fig. 3.**
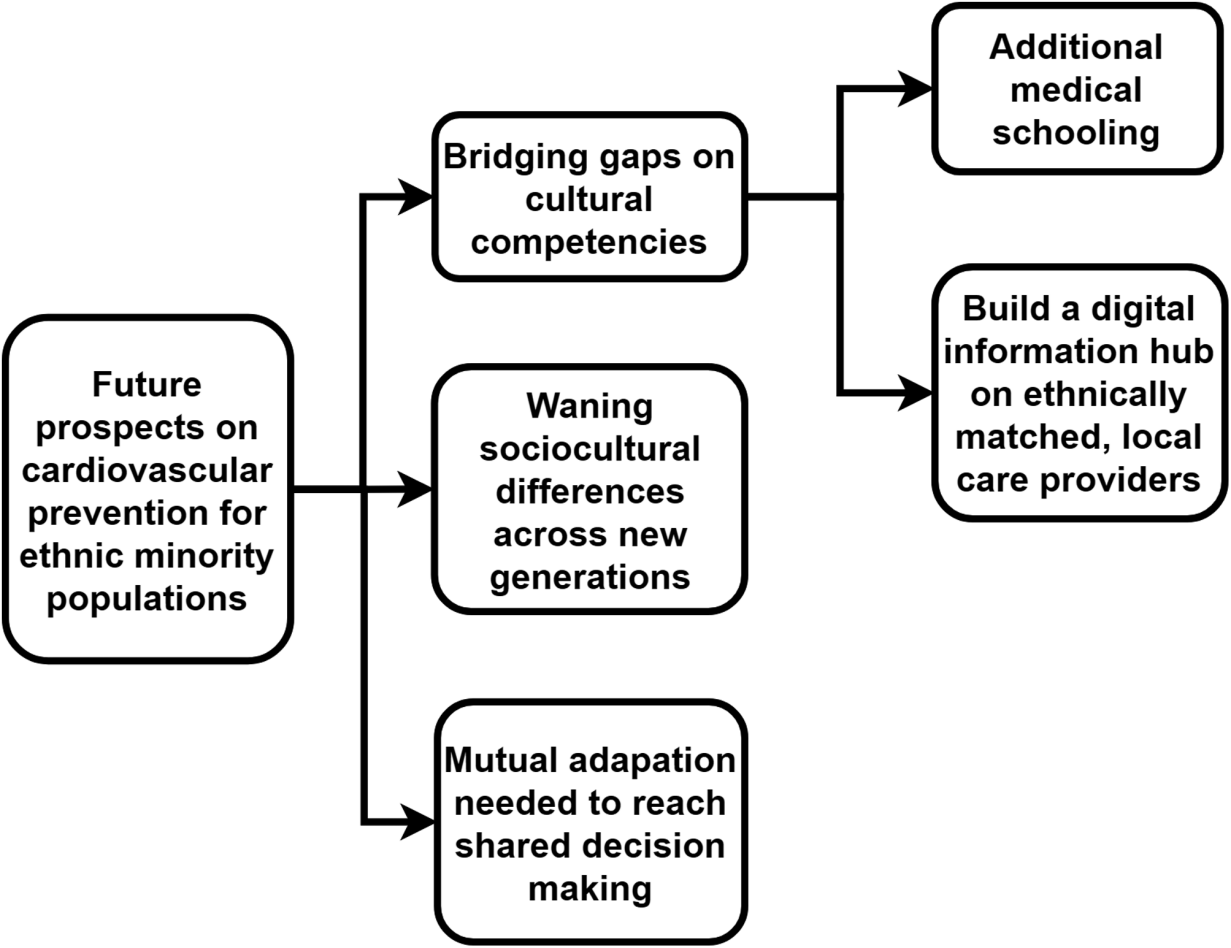
Thematic analyses key theme 3.

We interviewed 24 GPs (Table 1), with experience ranging from 1 to 44 years, with an average of 17 years. GPs’ ages ranged from 34 to 72 years. Overall, 46% worked in a group practice and 63% in an urban setting. GPs generally recognized ethnicity as a relevant determinant of CVD risk and sociocultural differences as an important aspect of treatment. However, opinions on the relevance of these factors to cardiovascular prevention varied substantially. Figures 1–3 show the main themes and subthemes.

**Table 1.** Characteristics of participating GP interviewees (*n* = 24).

Characteristics	Number (%)
Sex	
Male	12 (50)
Age	
<40 years	6 (25)
40–50 years	4 (17)
>50 years	14 (58)
Ethnicity	
European	20 (84)
Middle Eastern	2 (8)
South Asian	2 (8)
Years as a GP	
0–15 years	11 (46)
>15 years	13 (54)
Practice location	
Urban	15 (63)
Rural	9 (37)
Practice setting	
Small scale (solo/duo practice)	11 (46)
Group practice (≥3 GPs)	11 (46)
Locum GP (diverse practice experience)	2 (8)

### Defining the role of ethnicity in CVD risk assessment, communication, and treatment

#### Attitudes towards the role of ethnicity in cardiovascular prevention

GPs often regarded an ethnic minority background as a distinct phenomenon when providing CVD prevention. However, they preferred an individual approach to avoid generalization or stigmatization based on ethnic background.

I think that culture and ethnicity deserve a place in the consultation, but the individual should always be seen independently from the group. So you have to be careful about that; you can ask about things and that can be of added value, but you can never just assume that because someone has a certain background, certain things are going on.—GP 10 (female, 34)

However, low SES was felt to have more impact on overall cardiovascular risk through unhealthy lifestyle behaviours than genetic factors related to ethnic background. Regarding the current Dutch cardiovascular prevention guideline, some GPs indicated that ethnicity plays a minor role in CVD prevention. Others felt that more emphasis should be placed on ethnicity, as there are clear differences in CVD risk between ethnic groups, which should be recognized despite the current social restraints in addressing ethnicity.

Patients and doctors may experience [reference to black people] as problematic. But in some cases, it is important, just like you have to write down BRCA-gene in your medical records. So yes, I think we should be able to separate this [the medical relevance and the social discussion] and think: this is simply medically relevant.—GP 20 (female, 38)

#### Relevance of ethnicity for risk or assessment

GPs’ views on the potential impact of ethnicity on overall CVD risk varied substantially. While some thought ethnicity was irrelevant, others viewed it as an independent CVD risk factor. In addition, several GPs mentioned an increased prevalence of uncontrolled hypertension among patients of African descent, requiring specific antihypertensive medication. Similarly, interviewees reported an increased risk of diabetes and worse CVD prognosis in patients of South-Asian Surinamese origin.

Two years ago, a lady of 36 years old died of myocardial infarction, South-Asian Surinamese, she was. How often does that happen to someone of Dutch origin? Never, right?—GP 13 (male, 40)

This worse CVD prognosis in patients of South-Asian Surinamese origin could result in a lower screening threshold (e.g. screening of blood glucose level) when patients presented themselves with potentially related complaints.

So I’m also more inclined to perform more diagnostic testing, so, for example, to check glucose as well, (…) if someone comes in with a fungal infection and I do [increase diagnostics], I check their glucose straight away and sometimes it does turn out to be diabetes. Purely based on… well, if you [the interviewer] came in with a fungal infection, I wouldn’t normally check your glucose, but if you were Hindustani, I would have.—GP 12 (female, 47)

Some GPs who viewed specific ethnic backgrounds as an independent CVD risk factor ended up operationalizing ethnicity for CVD risk prediction intuitively in the absence of validated risk prediction models for these groups.

#### Sociocultural differences influencing cardiovascular prevention

GPs thought that providing cardiovascular prevention generally was more difficult for ethnic minorities than for the majority population. The most important barriers they experienced were finding mutual understanding and addressing patients’ different beliefs, values, and behaviours. GPs felt unfamiliarity with these cross-cultural aspects could negatively affect patients’ trust in their advice or treatment. This lack of knowledge about customs was also thought to hinder appropriate advice on physical exercise or diet. This was generally relevant since obesity constituted a substantial part of lifestyle-related risk factors.

When a Dutch person has a high LDL, then I start by mentioning eating cheese. But that would probably not be appropriate for someone with a Turkish background. […] So, food habits… No, I am quite unfamiliar with those.—GP 17 (male, 65)

Differences in the role food plays across different cultures were also mentioned. GPs acknowledged the importance of understanding the cultural importance of dietary customs since lifestyle-related advice will be futile if they remain unaddressed.

Because the question is whether the function of food is merely to stop feeling hungry or whether it has a totally different function, especially in non-Dutch cultures. In Eritrean patients, for instance, it has the function of hospitality and sharing, to be able to share what you have and to be welcoming.—GP 20 (female, 38)

GPs felt that if patients’ interpretation of the cause of chronic conditions like hypertension differed from the biomedical perspective, this could negatively impact medication adherence. For example, they mentioned that some patients stopped taking their antihypertensive medication during holidays, as these patients believed stress was the cause of their hypertension.

And then they go to Suriname for a few months, and they say: “Well, I never take my medication there because I never experience stress over there, so I do not need them.” And then they return with an astonishingly high HbA1C and a hypertensive crisis.—GP 24 (female, 41)

GPs mentioned that taking more time is necessary to truly understand their patients and their perceptions amidst all potential barriers.

Well, at the next consultation, it [her blood pressure and blood glucose] still had not really improved. And then, I took the time to talk to her about how she experienced her obesity. I even involved an official interpreter. And then, very surprisingly, she said: “In the Congo, it is a beauty ideal. Yes, we women try very hard to be chubby, especially with big breasts and big buttocks, which are very nice for men. And if you don’t have those, then you are not part of the group.”—GP 8 (male, 72)

### Barriers and potential solutions in cardiovascular prevention

#### Language barriers

All GPs mentioned language barriers as a limitation, often leading to longer consultations. Solutions mentioned for language barriers included simplification and asking patients to bring a family member to translate. The potential benefit of family members taking part in the consultation was an increased understanding of their sociocultural context, facilitating tailored advice on lifestyle behaviour change:

I felt the dynamic with a family member was somehow more relaxed. It just makes the situation more accessible. And then they know about everything too, so they can help with lifestyle stuff.—GP 4 (female, 30)

However, family members were often children, potentially complicating conversations with a more personal context. Moreover, GPs felt that information could be lost as family members sometimes refrained from translating information fully. Despite this, official interpreters were seldom consulted, as this was both time-consuming and costly. In most areas, there was no funding to reimburse the costs of the interpreter telephone line.

#### Harmonization between sociocultural factors and lifestyle interventions

GPs mentioned the importance of achieving shared decision-making. Sometimes this required spending more time on nonmedical subjects, such as family or migration history.

It is a form of investment to talk about other things sometimes. Not just about their behaviour, but, for instance, asking about their grandchildren; things like that. That way you invest in the relationship, which may help them trust you a bit more as their doctor. Yes, it does take a lot of time, but it is worthwhile.—GP 3 (female, 55)

Some of the interviewed GPs attained insight into cultural customs and health perceptions in collaboration with nurses from different ethnic backgrounds working in their practice and experienced beneficial effects on patient trust.

You know, patients really trust the practice nurse because they can communicate with her in their own language, and yes, I think that makes them feel more heard.—GP 11 (female, 36)

#### Culturally sensitive interventions to improve guidance on lifestyle behaviour change

As general lifestyle advice may not be appropriate for everyone, some GPs mentioned referral to paramedics who had developed lifestyle interventions for individuals with specific cultural backgrounds to increase their uptake and yield.

One physiotherapist […], noticed a couple of years ago that it was really difficult or even impossible to get some men to exercise, so he set up a dancing group for Moroccan men. He really made an effort to search for something that does work.—GP 2 (female, 62)

Some GPs actively tried to balance the biomedical and cultural explanatory models. This would, in turn, strengthen the shared decision-making, for instance, on medication adherence.

I always say, when you already have slightly stiff arteries—which can cause high blood pressure—and on top of that you experience stress, it only gets worse. […] But I always try to meet them halfway. In any case, I always try to involve the biology of it, hoping that next time they will use their medication. […] I sometimes ask them to bring a blood pressure monitor to Suriname or ask them to have their blood pressure measured by a doctor there of a family member. Sometimes it helps that they can simply see for themselves how their blood pressure is there.—GP 24 (female, 41)

### Future prospects on cardiovascular prevention for ethnic minority population groups

Prospects of the implication of ethnicity in future care varied considerably. Most GPs mentioned that attention to ethnic differences should be increased, and differences should be further explored to provide appropriate CVD prevention. GPs expressed a preference for additional postgraduate education. They felt that differential lifestyle behaviour guidance over so many ethnicities might become too detailed for the CVD prevention guideline, which was already perceived to be rather lengthy. Other suggestions to improve lifestyle intervention compliance among ethnic minorities included using a list of care providers specialized in ethnic minority groups to give patients a choice for a programme that corresponds with their customs and preferences.

If there were, say, a website where care providers […] with a certain cultural background can profile themselves, then you would not have to search as much. Then you could just say: here is a dietician with Indian roots, would you prefer that?—GP 10 (female, 34)

Conversely, some GPs felt that further integration by the younger migrant generations was the only viable route to overcoming intercultural differences. Like many GPs mentioned, second- and third-generation migrants were generally more influenced by Dutch culture than the first generation, which facilitated mutual understanding and lifestyle guidance. Other GPs mentioned that adaptation ought to be mutual. Both care providers and migrant patients should try to familiarize themselves with the other culture to reach mutual understanding and shared decision-making in CVD prevention.

Of course we can ask those people [ethnic minorities] to adapt to the Dutch values and culture, sure, I support that. […] But I also believe that the other party, the care providers, should equally familiarise themselves with the habits and values of those people. Mutual respect.—GP 21 (male, 50)

## Discussion

Ethnicity was broadly recognized as a relevant determinant of increased risk for CVD for most minority groups. While GPs were aware of sociocultural differences across migrant populations, they emphasized providing personalized care within a broader context, focussing on the individual situation and needs. The perceived increased CVD risk for specific ethnic groups often led to a higher propensity for earlier risk factor screening in individuals from these populations. Furthermore, GPs felt that the ability to arrive at a shared view was impeded by language barriers and differences in sociocultural perceptions and preferences, making it difficult to assess whether their guidance was appropriate and attainable. Suggested solutions included involving family members in the consultation and ethnically matched care providers (i.e. nurses), familiarizing themselves with perceptions and cultural customs and adapting lifestyle interventions to patients’ cultural backgrounds to increase the appropriateness of their lifestyle guidance. GPs expressed a need for continuing medical education on culturally sensitive care and reimbursement of telephone interpreting services.

Overall, GPs appeared hesitant to use ethnicity as an independent determinant of CVD risk. Some felt there was an insufficient evidence base for such an approach. In line with previous research, others felt that knowledge of the patient’s ethnic group should never be substituted for a personalized approach based on one’s unique personal values and context-related risk factors.^26,27^ The importance of an individual approach within a cultural context was highlighted by research among British GPs.^28^ It demonstrated GPs’ feelings of disempowerment and apprehension when confronted with an increasingly ethnically diverse population and showed that they felt they were falling short in providing culturally sensitive care. They expressed that focussing too much on patients’ cultural backgrounds and perceiving cultural competence as a skill may even fuel doctors’ uncertainty and feelings of insufficiency, to the detriment of patient care.^28^

While some GPs questioned whether differences between ethnic groups were even present, others were convinced that quality of care would be insufficient without paying attention to ethnicity-associated factors, including increased overall CVD risk, cultural preferences, and customs. This amounts to a lack of consensus on the importance of ethnicity in individual consultations^27^ and, likely, to GPs’ perceived feelings of competence in dealing with potential ethnic differences. This lack of consensus on the relevance of ethnicity may partly be explained by its complex link with culture, SES, and genetics, and how much importance GPs attribute to these aspects. For example, as GPs mentioned, cultural differences and low SES might impede some patients’ ability to adopt a healthy lifestyle, while genetic predisposition may influence their susceptibility to develop certain diseases.

Some GPs who used ethnicity as an additional yet unquantified weight for risk classification mentioned the need for ethnic-specific CVD risk calculators. The current Dutch CVD prevention guideline states that there is insufficient evidence for risk reclassification based on ethnicity but does recommend taking on a proactive attitude on risk assessment among ethnic minority populations.^29^ Contrary to the Dutch guideline, the European Society of Cardiology (ESC) CVD prevention guideline suggests using ethnic-specific CVD risk conversion factors and emphasizes the need for additional research on CVD risk recalibration using ethnicity.^30^ Our study underscores this need for research. Ideally, as the impact of ethnicity may differ across regions,^30^ further research should be country specific to optimally fit national guideline recommendations.

Our study is in accordance with an earlier qualitative study among GPs in the Netherlands, where differences in explanatory models and cultural differences in exercising could lead to feelings of inappropriate provision of cardiovascular prevention.^17^ Moreover, some GPs felt that the CVD prevention guideline should be more proactive towards acknowledging ethnic differences and challenges associated with guidance and treatment for minority groups. There have been accounts of GPs mentioning different perceptions of sub-Saharan African patients regarding the origin, potential impact and treatment of hypertension.^31^ In our study, familiarity with these different explanatory models were perceived to produce favourable results. For instance, asking patients to measure their blood pressure while being on holiday in their country of origin was perceived as an effective way to increase hypertension awareness and medication adherence in patients of African descent. Previous research demonstrated that paying attention to cultural differences concerning hypertension has beneficial effects on adherence to lifestyle advice and blood pressure levels.^32^ Despite a lack of consensus on its relevance in clinical care, GPs in our study commonly mentioned that specific advice per ethnic group would be challenging to incorporate into an already lengthy CVD guideline. They mentioned that continuing medical education on culturally sensitive communication and interventions would be well appreciated. Cultural sensitive communication has many similarities with patient-centred communication, a vital aspect of GP training and care.^33^ For example, both require building trust, checking whether patients understood the provided information and managing expectations. Intercultural differences necessitate these factors even further as differences in norms and values between the doctor and patient may challenge interpersonal aspects of communication. As previous researchers suggested, integrating intercultural communication into patient-centred communication can improve health care quality as they share similar goals.^33^ In our study, GPs expressed faith in the beneficial effects of culturally tailored care on disease prevention and the resulting increase in adherence to lifestyle intervention and medication. The expressed need for reimbursement and readily available official interpreters is corroborated by previous research; language barriers can enlarge cultural barriers and diminish access to the health care system, provision of lifestyle advice, and ability to understand patients’ perceptions of the origins, impact and optimal treatment of CVD risk factors, potentially contributing to CVD ethnic health disparities.^34,35^

### Limitations

Most interviews were held through video conferencing due to the COVID-19 pandemic. This may have led to the loss of nonverbal communication. However, we are confident this did not have a substantial impact, given the open and frank conversations we experienced with these interviewees. Potential bias could have occurred as less than 30% of the approached GPs took part in our study, which may be due to specific interest in the research topic. However, some GPs mentioned openly during the interview that they had not given the factor of ethnicity in cardiovascular prevention much thought. Another potential limitation is that some of the questions in our interview guide could have had a more open-ended nature, which may have impeded elaborate answers by participating GPs. Nonetheless, we encouraged GPs to substantiate statements by asking follow-up questions to truly understand their perspective. Participating GPs were given the opportunity to reflect on the transcripts or summaries of interviews, but this was rarely done, mostly due to perceived time constraints. In this study, we focussed on the experiences of GPs. However, as was emphasized by many interviewees, the role of Dutch practice nurses in cardiovascular prevention programmes has become more prominent over the last years.^36^ Therefore, their views and experiences may yield valuable additional knowledge in future studies.

## Conclusion

Dutch GPs have differing views on the role of ethnicity in evaluating and treating cardiovascular risk. Despite these differences, they emphasized the importance of a personalized and culturally tailored approach during patient consultations and expressed a need for continuing medical education. Additional research on how ethnicity influences CVD risk may strengthen cardiovascular prevention in the increasingly diverse primary health care population.

## Supplementary material

Supplementary material is available at *Family Practice* online.

cmad030_suppl_Supplementary_File_S1

cmad030_suppl_Supplementary_Checklist

## Data Availability

The data underlying this article cannot be shared publicly due to the privacy of individuals participating in the study. The data will be shared on reasonable request to the corresponding author.
