# Supplementary material for: General practitioners’ views on cardiovascular prevention for ethnic minorities—a qualitative study in the Netherlands
Source: Fam Pract. 2023 Mar 30;41(3):340–8. doi: 10.1093/fampra/cmad030 (PMC11167987; doi:10.1093/fampra/cmad030)
Supplement: cmad030_suppl_Supplementary_File_S1 [file cmad030_suppl_supplementary_file_s1.docx]

**Interview guide - General practitioners' views on cardiovascular prevention for ethnic minorities**

**Consent to participation and recording**

**Introduction general practitioner (GP)**:
Age, years of experience, type of practice, composition of ethnic backgrounds within practice, own ethnicity.

General:
- Does prevention belong to the responsibilities of the GP? Where is the line between the responsibility of the patient and that of society, and where does the responsibility of the GP begin?
- Do you think patients benefit from lifestyle interventions?

**Ethnic differences in cardiovascular risk management**

Risk prediction
 - Do you consider ethnicity as an independent factor in your assessment of the risk of cardiovascular disease? If so, in which ways? Do you apply this risk in practice, for example by multiplying the risk by a certain factor for a high-risk group? Does this differ across ethnicities? Which factors play a role in this?
- Do you feel that the guideline is appropriate when it comes to CVD risk estimation in ethnic minorities? Would you use a risk calculator in which ethnicity is part of the overall CVD risk assessment?
- Are you aware of the different habits in other cultures and do you refer to these when providing lifestyle advice? Do you observe beneficial effects from such an approach? How do you match culturally sensitive questions to the life of patients?
- Do you study the explanatory model of patients for the origin of disease (for instance, hypertension)? Do you use this as a basis for shared decision-making?
- Would you feel a need for examples of culturally sensitive communication of cardiovascular risk? For example, information about various common explanatory models for cardiovascular disease and risk factors within different groups? If yes, do you feel that examples of culturally sensitive communication should be incorporated in the guideline or would postgraduate education be a more appropriate place to learn on this subject?
- Do you ask patients about their ethnicity, and if so, how do you ask about this and whom? How could one gather information about ethnicity in a GP practice without creating feelings of awkwardness or negatively affecting the relationship?
- Does ethnicity play a role in your choice of prescribed medication (for example, antihypertensives)? How do you make sure to stay aware of this, during daily practice? Might registration of ethnicity be a helpful tool (an alert, for instance), or could it be useful to have documents to check whether the correct medication has been prescribed?
- Is it justified to distinguish patients based on their ethnicity if this improves the medical results? Does the relevance outweigh the risk of discrimination?

Limitations
 - Which barriers do you experience with regards to cardiovascular risk prevention in ethnic minorities?
- Do different ethnic backgrounds complicate the process of providing and monitoring lifestyle advice? How significant is the contribution of different explanatory models to this? How could mutual understanding be improved? How does the failure to follow your advice affect your motivation to sustain a culturally-sensitive approach in patient care?
- What is the role of language? How do you cope with language barriers? (family members as interpreters? Using a telephone interpreting service? Engage practice nurses with the same ethnic background? Other solutions?)
- What about dietary advice? Do you feel that knowledge of the dietary habits of ethnic minorities is required to provide appropriate advice/do your job as general practitioner? Is there a benefit to refer to or collaborate with dieticians with specific knowledge about the dietary habits of different ethnic minorities?

Potential solutions
 - Have you found any solutions yourself to overcome these barriers/to address these challenges? Can you give an example?
- Can you think of examples of successful risk communication or lifestyle advice in the provision of cardiovascular risk management for ethnic minorities? (If this involved an ethnically matched practice nurse, what made it so effective?)
- Which suggestions for improvement of cardiovascular prevention for ethnic minorities do you have for your practice nurse and vice versa: what can they teach you?
- Are there any local initiatives to adopt a healthy lifestyle for ethnic minorities in your region? Do you see a function for these?
- Do you think it might be useful to engage with community leaders of ethnic minority groups to learn about healthcare demands, views on health and disease, lifestyle routines, and culturally specific preferences on self-management?
